# The highs and lows of lifting loads: SPM analysis of multi-segmental spine angles in healthy adults during manual handling with increased load

**DOI:** 10.3389/fbioe.2024.1282867

**Published:** 2024-01-25

**Authors:** Jasmine K. Proud, Alessandro Garofolini, Kurt L. Mudie, Daniel T. H. Lai, Rezaul K. Begg

**Affiliations:** ^1^ Institute for Health and Sport (IHES), Victoria University, Melbourne, VIC, Australia; ^2^ Land Division, Defence Science and Technology (DST), Melbourne, VIC, Australia; ^3^ College of Sport, Health and Engineering, Victoria University, Melbourne, VIC, Australia

**Keywords:** biomechanics, manual handling, statistical parametric mapping, assistive technology, spine angles, inertial measurement units

## Abstract

**Introduction:** Manual handling personnel and those performing manual handling tasks in non-traditional manual handling industries continue to suffer debilitating and costly workplace injuries. Smart assistive devices are one solution to reducing musculoskeletal back injuries. Devices that provide targeted assistance need to be able to predict when and where to provide augmentation via predictive algorithms trained on functional datasets. The aim of this study was to describe how an increase in load impacts spine kinematics during a ground-to-platform manual handling task.

**Methods:** Twenty-nine participants performed ground-to-platform lifts for six standardised loading conditions (50%, 60%, 70%, 80%, 90%, and 100% of maximum lift capacity). Six thoracic and lumbar spine segments were measured using inertial measurement units that were processed using an attitude-heading-reference filter and normalised to the duration of the lift. The lift was divided into four phases weight-acceptance, standing, lift-to-height and place-on-platform. Statistical significance of sagittal angles from the six spine segments were identified through statistical parametric mapping one-way analysis of variance with repeated measures and *post hoc* paired t-tests.

**Results:** Two regions of interest were identified during a period of peak flexion and a period of peak extension. There was a significant increase in spine range of motion and peak extension angle for all spine segments when the load conditions were increased (*p* < 0.001). There was a decrease in spine angles (more flexion) during the weight acceptance to standing phase at the upper thoracic to upper lumbar spine segments for some condition comparisons. A significant increase in spine angles (more extension) during the place-on-platform phase was seen in all spine segments when comparing heavy loads (>80% maximum lift capacity, inclusive) to light loads (<80% maximum lift capacity) (*p* < 0.001).

**Discussion:** The 50%–70% maximum lift capacity conditions being significantly different from heavier load conditions is representative that the kinematics of a lift do change consistently when a participant’s load is increased. The understanding of how changes in loading are reflected in spine angles could inform the design of targeted assistance devices that can predict where and when in a task assistance may be needed, possibly reducing instances of back injuries in manual handling personnel.

## 1 Introduction

The rehabilitation, over-employment, re-education and inquiry of serious workplace injuries costs the Australian economy AU$28.6 billion per year (Safe Work Australia, 2023a). Serious workplace injuries including traumatic joint, ligament, muscle and tendon injuries make up 36.6% of injury claims (Safe Work Australia, 2023b). While the agriculture, forestry, fishing industry had the highest injury frequency rate (10.9 serious claims per million hours worked), it was the healthcare and social assistance industry that had the highest number of serious claims at 18.9% (Safe Work Australia, 2023b) and the accommodation and food services industry that has the highest work related injury rates at 56.4 per 1,000 workers (Safe Work Australia, 2023a). Both of these industries would be considered non-traditional manual handling industries, however lifting, pushing, pulling or bending, which are manual handling movements, were the most common cause of workplace injury in Australia with 24.1% of all serious claims (Safe Work Australia, 2023a).

Assistive devices that can support workers are one solution for preventing workplace injuries and improving productivity. Targeted assistive devices have the ability to minimise the impact of contributing risk factors such as lifting above a person’s capability (Rosenblum and Shankar, 2006; Savage et al., 2012), lumbar spine hyper-flexion/extension (Ferguson et al., 2004; Neumann, 2009) and from-the-ground lifting (Ngo et al., 2017). For assistive devices to be successful they rely on intelligent algorithms and informed design (Zaroug et al., 2019; Proud et al., 2022). A deeper understanding of the mechanism the spine uses at multiple levels to compensate for increased loading could provide information about the spine’s contribution at different stages of the lift; this could be useful for targeted assistive devices to know when and where assistance is needed.

There are mixed results of the effect of load on the lumbar and thoracic spine, depending on what segment is being studied and what phase of the lift is being analysed. Some studies reporting a significant decrease in angle with an increase in load (Scholz et al., 1995; Mirka and Baker, 1996; Melino et al., 2014; West et al., 2018); others report a significant increase in angle with an increase in load (Van Der et al., 2000; Song and Qu, 2014a; Song and Qu, 2014b; Elsayed et al., 2015). This could be because studies into kinematic changes for manual handling tasks used discrete features [e.g., peak, minimum, mean, range of motion (ROM)] (Allread et al., 1996; MacKinnon and Li, 1998; Gatton and Pearcy, 1999; Davis and Marras, 2000; Zhang et al., 2003; Song and Qu, 2014a; Song and Qu, 2014b; West et al., 2018) and/or time periods (e.g., start, middle, end) (Scholz et al., 1995; Allen et al., 2012; Song and Qu, 2014a; Song and Qu, 2014b; Melino et al., 2014; Antwi-Afari et al., 2018; West et al., 2018) to explore the relationship between increased load and spine kinematics. The most commonly used discrete features were peak, mean and ROM and the variables analysed were trunk/lumbar angle, angular velocity and acceleration. Significant correlations between increased load and these discrete features were found in the literature (Scholz et al., 1995; Allen et al., 2012; Song and Qu, 2014a; Song and Qu, 2014b; Melino et al., 2014; West et al., 2018), such as a significant increase in trunk extension angle at the end stage of the lift (Song and Qu, 2014a; Song and Qu, 2014b), decreased thoracic extension angle (T7) at the end stage of the lift (Allen et al., 2012; West et al., 2018) and increase lumbar extension angle (L5) (Allen et al., 2012).

Existing literature is limited to reporting kinematic changes at discrete points within the lift cycle, and it is not known the effect of loading during the entire lift. Additionally, a study of six points along the spine using multiple standardised load conditions has not been previously performed. It is therefore uncertain at what discrete point(s) in a lift the different segments of the spine are affected by load. This information is vital for assistive devices to provide targeted support. Looking at the complete time series of the lift was vital to understanding the intricacies of where and how the task was affecting spine kinematics.

**FIGURE 1:**
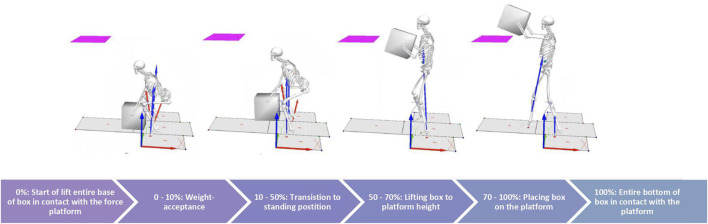
Phase of lift cycle (ground to platform lift).

The aim of this study was to describe how an increase in load impacts spine kinematics during a ground-to-platform manual handling task. The ground-to-platform lift task involves four phases as shown in Figure 1: weight-acceptance, standing, lift-to-platform-height and place-on-platform. This research hypothesised that there would be a positive relationship occurring in the lift-to-platform-height phase (greater extension with increased load), a negative relationship in the weight-acceptance phase (greater flexion with increased load) and that these relationships would be seen at all levels of the spine.

## 2 Methodology

### 2.1 Participant information

An analysis of variance (ANOVA) repeated measures, within factors power analysis was performed prior to commencement of experiments (f = 0.25, α = 0.05, rho = 0.25) that indicated a sample size of 25 would be sufficient to produce a power above 0.80 (Faul et al., 2007). Thirty-two healthy participants between 18–40 years were recruited via flyers and word-of-mouth from the Victoria University student population for this study. Therefore, manual handling experience was widely varied within the participant cohort. Prior to participation in the trial, participants were required to provide written confirmation of informed consent, fill out a health survey and be free from musculoskeletal injury and any illness, disease or condition that put them at risk during intensive exercise. All participants were phoned the day before testing to enquire on their general health, answer any questions and informed to refrain for an intensive exercise prior to testing. Institutional ethical approval was received from the Victoria University Human Research Ethics Committee (HRE18-231).

### 2.2 Measurement equipment

All testing was performed at the Victoria University Biomechanics Laboratory. Nine-axis IMUs (ImeasureU, Vicon Motion Systems Ltd., Oxford, United Kingdom) were used to record acceleration (triaxial accelerometer ±16 g), angular velocity (triaxial gyroscope ±2000°/s) and magnetic field strength (triaxial magnetometer ±4900 µT) at a sampling frequency of 500 Hz. IMU trial data was recorded via the IMU Research app (ImeasureU, Vicon Motion Systems Ltd., Oxford, United Kingdom). Additionally, 12 Vicon cameras (Vicon Motion Systems Ltd., Oxford, United Kingdom) were used to record 18, 9 mm reflective markers attached to the IMUs (three to each sensor) recorded at 100 Hz.

In pilot studies it was found that an IMU placed at the base of the skull, as done in previous studies using a seven-segment model (Preuss and Popovic, 2010; Noamani et al., 2018) caused discomfort to participants during squat portion of the lift so the number of sensors was reduced to six. The IMU sensors were placed mid-way between the C7 -T3 (upper thoracic), T3-T6 (middle upper thoracic), T6-T9 (middle lower thoracic), T9-T12 (lower thoracic), T12-L3 (upper lumbar) and L3-S1 (lower lumbar) spinous process. The orientation was *Z*-axis in the anterior-posterior direction (anterior in the positive, posterior in the negative), *X*-axis in the medio-lateral direction (positive to the right, negative to the left) and *Y*-axis in the vertical direction (superior being positive, inferior being negative). Sagittal plane analysis would include information in the *Y* and *Z*-axes.

### 2.3 Testing procedure

A ground-to-platform lifting task was performed comprising of lifting a single crate with side mounted handles from the ground to a 1.4 m platform (Savage et al., 2012; Carstairs et al., 2017). This involved two procedures, 1) the first protocol determined the maximum load a participant can lift for a single repetition, and 2) the second involved lifting loads at seven conditions (20%, 50%, 60%, 70%, 80%, 90%, and 100% of their determined maximum lift capacity from protocol one in a quasi—randomised order. Both protocols were performed in a single day with a 20 min break given between protocol one and protocol two. All tests were conducted with two researchers, one being a qualified physiotherapist.

A 3-min warm up was performed followed by familiarisation with the task. This involved instruction on the squat lifting technique, practicing the lifting technique with the box (8 kg) and information about the criteria for a passable lift, such as placement of the box and technique. Prior to each lift, participants were asked to perform a small jump to align the IMU and Vicon data. Each lift was performed using a squat posture, the participants then extended to standing positions, the box was then lifted to the height required to place it on the platform and taking a step forward to a split stance posture, placing the box on the platform. Three minutes rest, or more if requested, was given between each lift to minimise the effect of fatigue.

The procedure to determine maximum lift capacity involved participants starting with a 10 kg box mass (8 kg box mass +2 kg weight plate) and completing the lifting task. The mass was then increased by 5 kg after every completion with correct technique (i.e., good posture) until the lift fails or technique deteriorates. Deterioration was characterised as, a change in posture (stooped position instead of squat position), inability to maintain symmetry in the lift (leaning to one side, twisting), needing to take more than one step in order to reach the platform or excessive hyper-extension of the lumbar spine where the line of the shoulders is posterior to the pelvis (Hamill and Knutzen, 2006). The mass was then lowered by 2.5 kg and attempted again. If completed this determined the participants maximum lift capacity or if failed the previous mass was the recorded maximum lift capacity.

Once a participant’s maximum lift capacity was determined, a quasi-randomised procedure was then performed to record spine kinematics at seven load conditions. Participants first lifted 100% of their maximum lift capacity three times. Each condition (percentage of maximum lift capacity: 20%, 50%, 60%, 70%, 80%, and 90%) was then lifted three consecutive times in a randomised order. The load was added to the box behind a screen that obstructed the participants view. Participants were informed whether the box was a heavy, medium or light weight prior to the first lift. Two participants were missing a spine segment of IMU data. Furthermore, due to the limitations of the box mass alone (8 kg) 18 participants were unable to perform the 20% condition and one was unable to perform the 50% maximum lift capacity condition. Due to the limitations of the minimum box weight and repeated measures statistics requiring balanced datasets, only six conditions (50% MLC to 100% MLC) and 29 participants were included in analysis.

The duration of the lift (0%–100%) was segmented into 4 phases (Figure 1). These phases were determined *a priori* and the percentage of the lift that the phases took was based on observation. The lift begins (0%) in the squat position with hands placed on the box and the entire base of the box in contact with the ground. Phase 1 (0%–10%) was the weight-acceptance phase, the external load was transferred from the ground to being held entirely by the participant. Phase 2 (10%–50%) was the standing phase, the participant transitioned from squat to standing position and the box was at waist height. Phase 3 (50%–70%) was the lift-to-platform-height phase, the participant lifted the box from waist height to the height required to clear the platform. Phase 4 (70%–100%) was the place-on-platform phase, the box was placed with its base coming into contact with the platform.

### 2.4 Data processing

The IMU data was down-sampled from 500 Hz to 100 Hz (down-sample function, factor of 5) with the purpose of being aligned with motion capture data for confirmation of start and end points of the lift. The data for each spine segment was aligned to the jump acceleration peaks prior to each lift and then divided into individual trials. It was found that over the approximate hour of recording for each participants %MLC protocol, there was a difference of one to 7 s of data (100–700 frames) between the spine segments. The data was divided into individual trials based on the vertical jump acceleration peak to the next vertical jump acceleration peak, each individual trial for each spine segment had the vertical acceleration peaks aligned. Once the lift data from the seven conditions was divided into its individual lift trials, the orientation of each trial was reordered to align with the north-east-down orientation. The acceleration, angular velocity and magnetometer data was passed through the attitude-heading-reference system fusion algorithm to estimate orientation (Sensor fusion and tracking toolbox, The MathWorks Inc., Natick, MA, United States). The attitude-heading-reference filter uses an indirect Kalman filter to output Euler angles. Only angles in the sagittal plane were analysed as this is where the majority of motion for a two-handed squat lift will occur. The IMU variable used for comparison was absolute angle around the *Y*-axis (flexion/extension motion). Static posture angles recorded during quiet standing were deducted from the spine angles, normalising the dataset to static posture.

### 2.5 Statistical analysis

**FIGURE 2:**
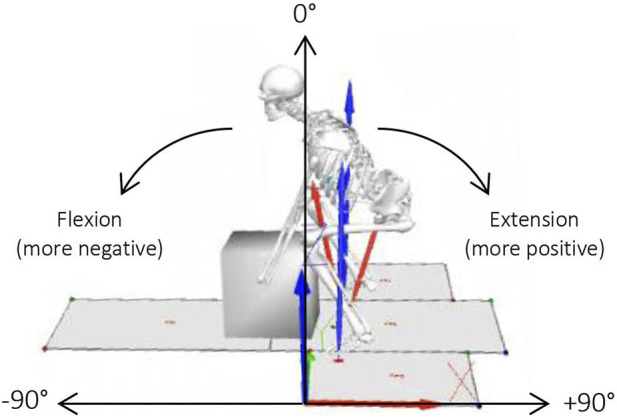
Direction of flexion/extension for sagittal angles.

The time from start to end of the lift was normalised to percentage (101 data points) (*x*-axis) and plotted against the mean (with standard deviation cloud) of each condition (percentage of maximum lift capacity) (*y*-axis) to observe any trends in the data for each spine segment. The normalised angle results are in the sagittal plane with reference to the global axis. The sagittal plane represents 0°, with flexion creating a more negative angle and extension creating a more positive angle (Figure 2). In order to see any significant differences between the continuous condition angles, statistical parametric mapping (SPM) techniques were used from the open-source spm1d-package (spm1d.org, T. Pataky) in MATLAB (The MathWorks Inc., Natick, MA, United States). The data was found to be not normally distributed using the SPM function (spm1d.stats.normality.anova1rm). Non-parametric one-way analysis of variance (ANOVA) with repeated measures (spm1d.stats.nonparam.anova1rm) and *post hoc* paired t-tests (spm1d.anova_posthoc, spm1d.stats.nonparam.ttest_paired) were used (Nichols and Holmes, 2002). For the discrete variables, peak extension, peak flexion and spine ROM, normality was confirmed using Shapiro-Wilks normality test and Q-Q plots. Statistical analysis was performed using one-way ANOVA with repeated measures and *post hoc* paired t-tests in MATLAB (The MathWorks Inc., Natick, MA, United States). Sex was not used as a factor, as it has been found that male and female participants have similar lifting techniques when the load is standardised via MLC (Sadler et al., 2013; Sheppard et al., 2016). The alpha level was set at 0.05 and Bonferroni corrected (0.05/15 = 0.0033) for the *post hoc* tests. For the one-way ANOVA, if significant (*p* < 0.05), the main effect of load on spine angles (F-values) and *p*-values for the six spine segments was recorded. For the *post hoc* analysis the mean for each variable and %MLC class was compared to one another and the phase in which the significant difference occurred was reported.

## 3 Results

Participants in this study consisted of 20 male and 9 females, with a mean age of 29.5 ± 5.6 years, a mean height of 1.77 ± 0.10 m and mean mass of 75.2 ± 12.7 kg. The 100% of maximum lift capacity loads lifted by the participants ranged from 20 kg to 60 kg (mean maximum lifting loads for males 43.8 ± 9.7 kg and for females 22.8 ± 4.0 kg), which was 55% ± 11% of the males body mass and 35% ± 9% of the females body mass. The 1.4 m platform height represented 78% ± 4% of the males body height and 82% ± 4% of the females body height.

### 3.1 SPM analysis

**FIGURE 3:**
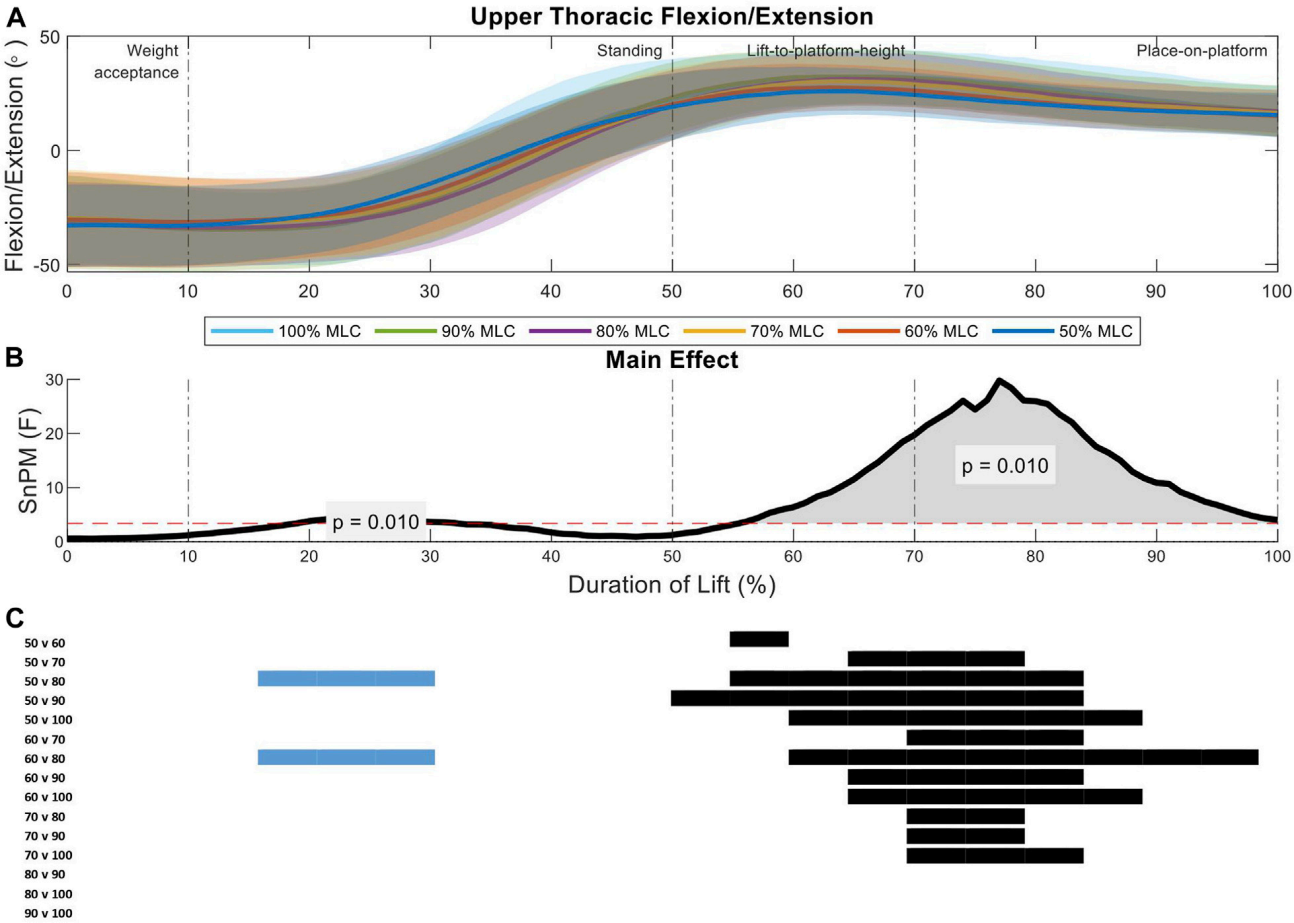
Upper thoracic angle pattern at standardised loads (%MLC). **(A)** Sagittal spine angle with SD clouds (1 SD) for six load conditions. **(B)** Time-dependent F-values of the SPM (main statistical test; analysis of variance) for all subjects (dashed red line; α ≤.05). **(C)** Post-hoc tests between six load conditions (*post hoc* results; α ≤.0033) and the phase of the lift where significance occurs. Black (positive) and blue (negative) bars span the region of the lift where significant differences were observed.

**FIGURE 4:**
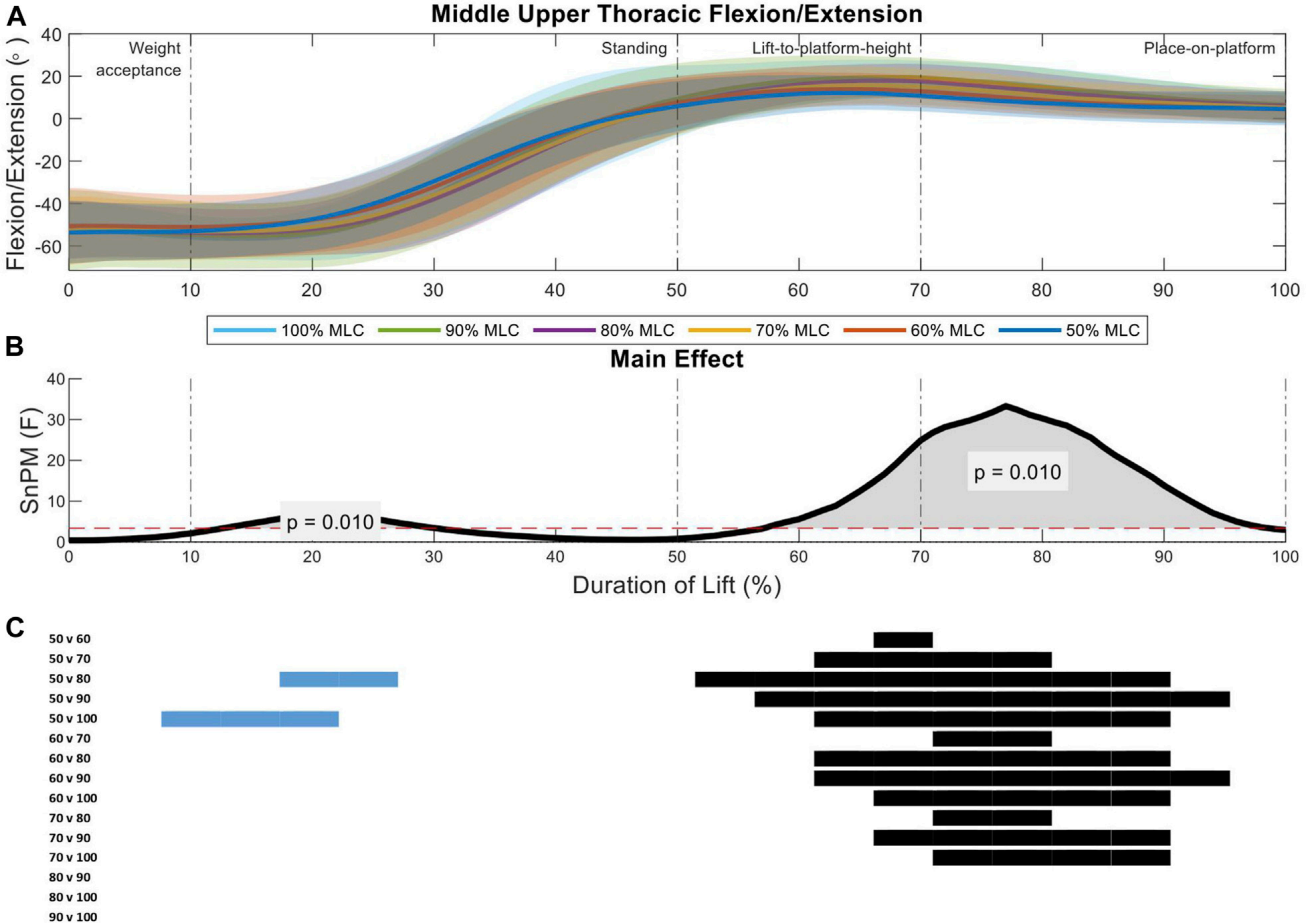
Middle upper thoracic angle pattern at standardised loads (%MLC). **(A)** Sagittal spine angle with SD clouds (1 SD) for six load conditions. **(B)** Time-dependent F-values of the SPM (main statistical test; analysis of variance) for all subjects (dashed red line; α ≤.05). **(C)** Post-hoc tests between six lond conditions (*post hoc* results; α ≤.0033) and the phase of the lift where significance occurs. Black (positive) and blue (negative) bars span the region of the lift where significant differences were observed.

**FIGURE 5:**
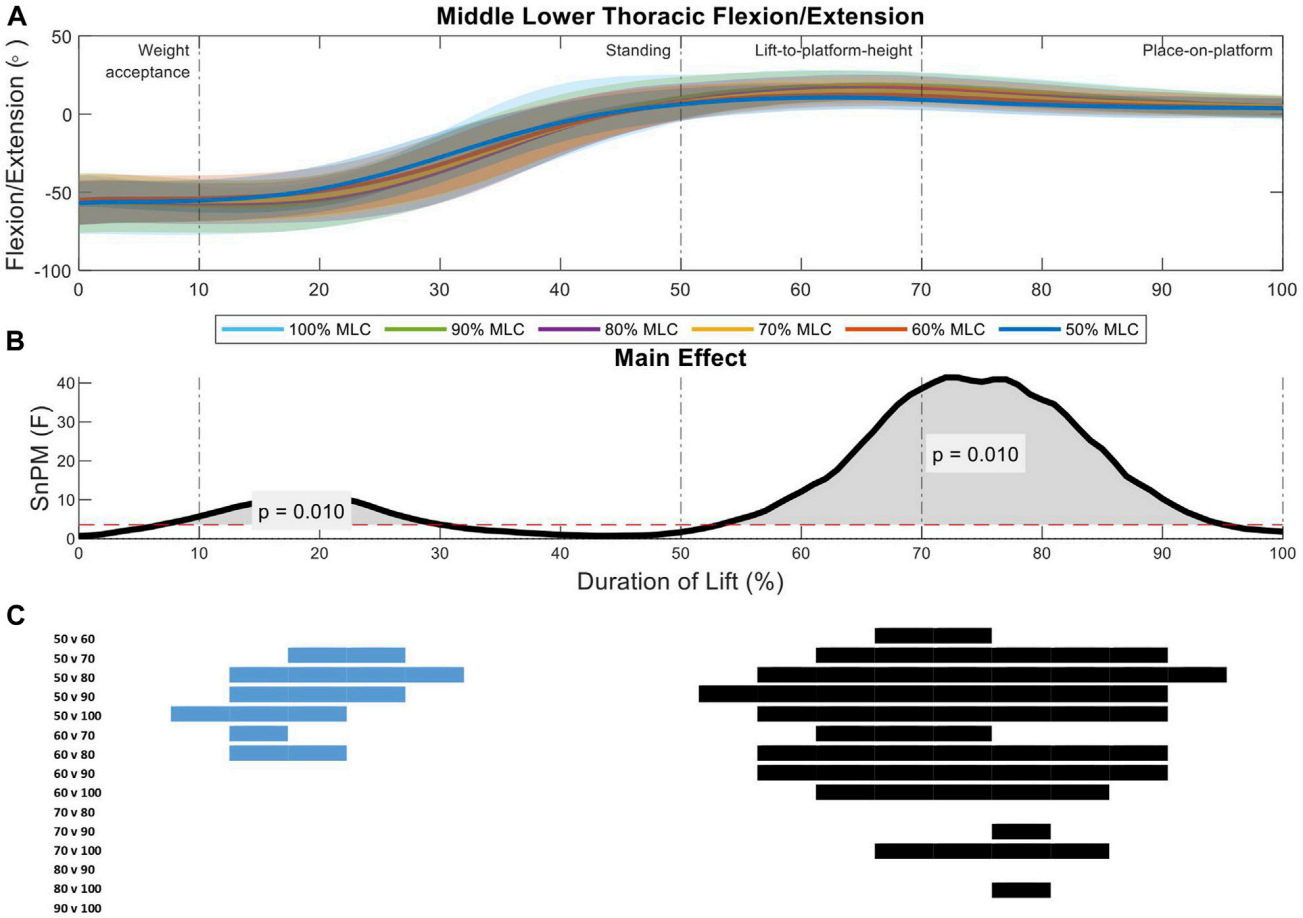
Middle lower thoracic angle pattern at standardised loads (%MLC). **(A)** Sagittal spine angle with SD clouds (1 SD) for six load conditions. **(B)** Time-dependent F-values of the SPM (main statistical test; analysis of variance) for all subjects (dashed red line; α ≤.05). **(C)** Post-hoc tests between six load conditions (*post hoc* results; α ≤.0033) and the phase of the lift where significance occurs. Black (positive) and blue (negative) bars span the region of the lift where significant differences were observed.

**FIGURE 6:**
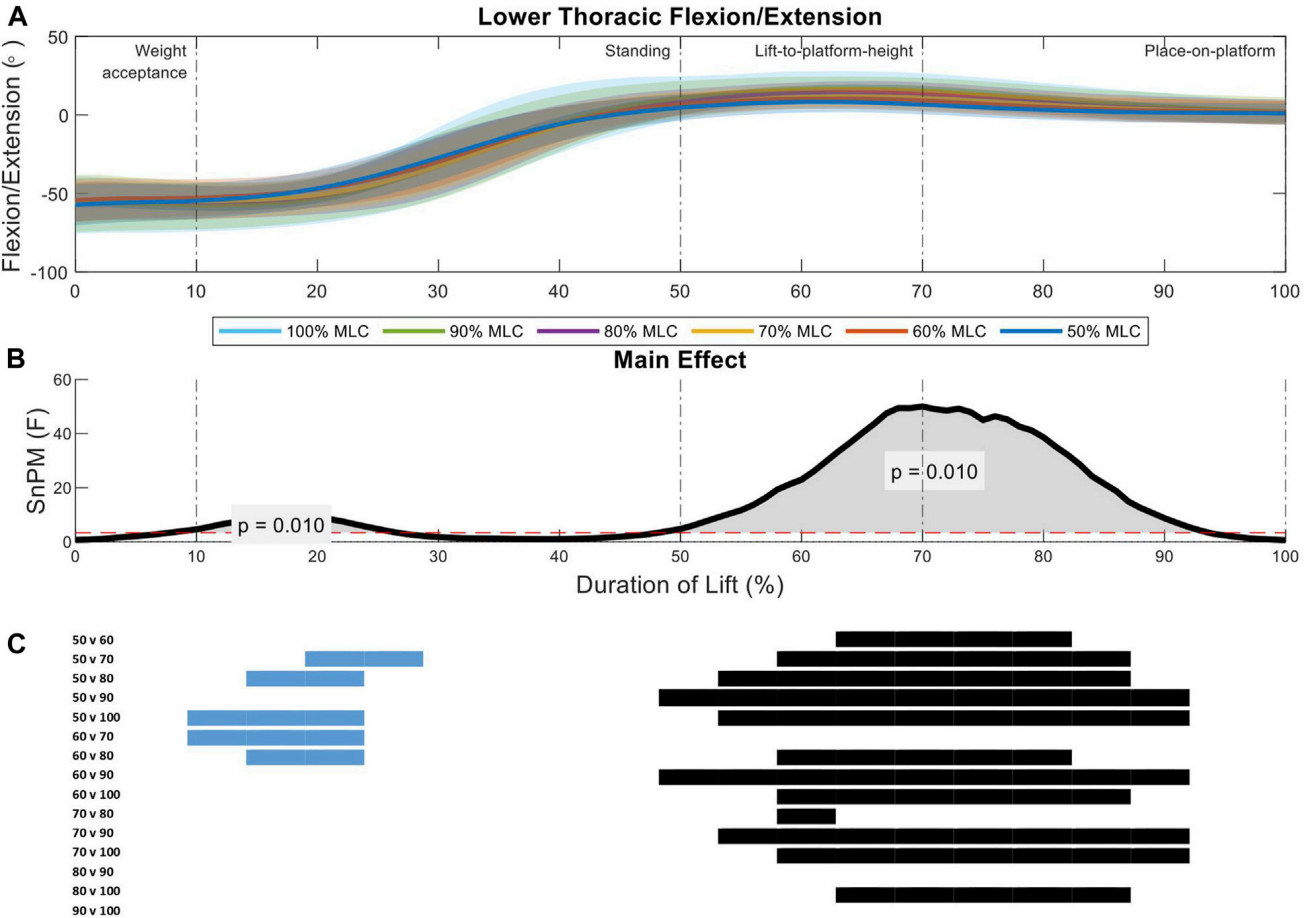
Lower thoracic angle pattern at standardised loads (%MLC). **(A)** Sagittal spine angle with SD clouds (1 SD) for six load conditions. **(B)** Time-dependent F-values of the SPM (main statistical test; analysis of variance) for all subjects (dashed red line; α ≤.05). **(C)** Post-hoc tests between six load conditions (*post hoc* results; α ≤.0033) and the phase of the lift where significance occurs. Black (positive) and blue (negative) bars span the region of the lift where significant differences were observed.

**FIGURE 7:**
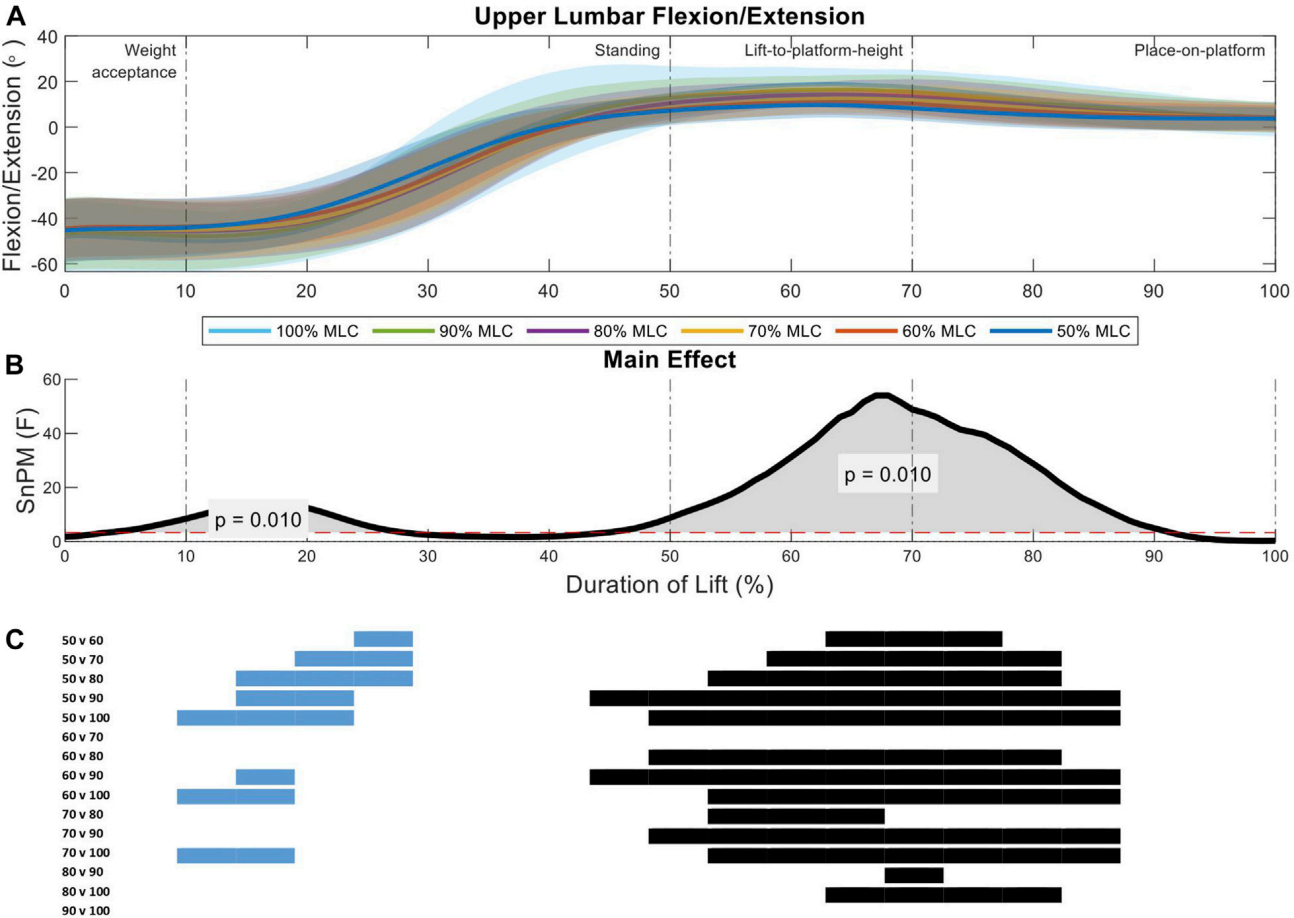
Upper lumbar angle pattern at standardised loads (%MLC). **(A)** Sagittal spine angle with SD clouds (1 SD) for six load conditions. **(B)** Time-dependent F-values of the SPM (main statistical test; analysis of variance) for all subjects (dashed red line; α ≤.05). **(C)** Post-hoc tests between six load conditions (*post hoc* results; α ≤.0033) and the phase of the lift where significance occurs. Black (positive) and blue (negative) bars span the region of the lift where significant differences were observed.

**FIGURE 8:**
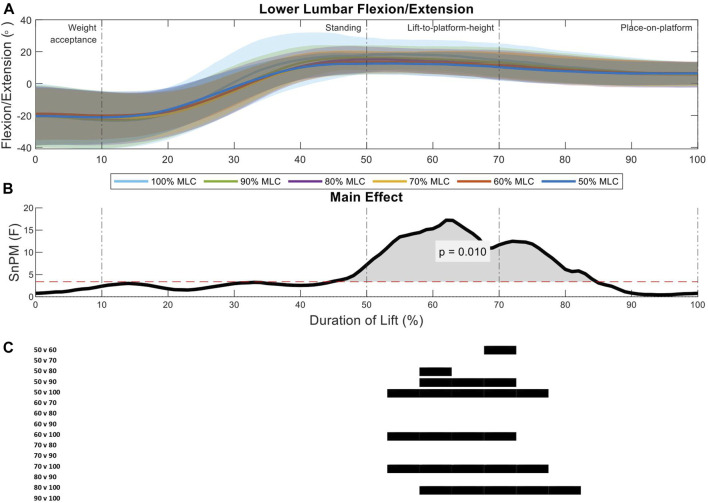
Lower lumbar angle pattern at standardised loads (%MLC). **(A)** Sagittal spine angle with SD clouds (1 SD) for six load conditions. **(B)** Time-dependent F-values of the SPM (main statistical test; analysis of variance) for all subjects (dashed red line; α ≤.05). **(C)** Post-hoc tests between six load conditions (post hoc results; α ≤.0033) and the phase of the lift where significance occurs. Black (positive) and blue (negative) bars span the region of the lift where significant differences were observed.

SPM analysis with repeated measures ANOVA showed a significant effect of load condition on the segmental spine angles for all spine segments (*p* = 0.01) (Figures 3B–8B). Two main regions of interest were found for all spine segments, the first occurring during the standing phase of the lift and the second occurring across the lift-to-platform height and place-on-platform phases of the lift. Post-hoc analysis showed that there was a negative relationship in the first region of interest, meaning that as the load increased, spine flexion increased (Figures 3C–8C). In the second region of interest there was a positive relationship, meaning as the load condition increased, spine extension increased (Figures 3C–8C). The majority of effect was in the comparisons between the lighter loads (50%–70% MLC) and in the comparisons between the 100% MLC condition and sub-maximal conditions. Spine extension (>0°) occurred in all segments of the spine during the lift-to-platform-height phase with larger extension caused by the heavier load conditions (Figures 3A–8A). The 90% MLC and 100% MLC load conditions did not show differences in *post hoc* comparisons, showing similar sagittal spine angle patterns (Figures 3C–8C).

Analysis of the upper thoracic sagittal spine angles between load conditions indicate two significant regions of interest (21%–33%, *p* = 0.01; 54%–98%, *p* = 0.01) (Figure 3B). During the standing phase of the lift (first region of interest) the 80% MLC spent longer in flexion when compared to the 50% and 60% MLC conditions. The greater F-values were seen in the lift-to-platform height and place-on-platform phases (second region of interest) (Figure 3B), where significant positive differences can be seen across all lighter load (<70% MLC) condition *post hoc* comparisons (Figure 3C). Furthermore, the heavier loads (80%, 90%, and 100% MLC conditions) had very similar peak extension angles showing no difference from each other (Figures 3A, C).

The middle upper thoracic sagittal spine angles analysis between load conditions revealed two significant regions of interest (14%–31%, *p* = 0.01; 57%–96%, *p* = 0.01) (Figure 4B). The first region of interest occurs in the standing phase of the lift, where the 50% MLC condition had less flexion that the 80% and 100% load conditions (Figures 4A, C). The second region had a larger main effect in the lift-to-platform height and place-on-platform phases (Figure 4B), where significant increased load conditions, increase spine extension across all load condition *post hoc* comparisons less than 70% MLC (Figure 4C).

Two significant regions of interest were shown in the analysis of the middle lower thoracic spine between load conditions (10%–35%, *p* = 0.01; 53%–94%, *p* = 0.01) (Figure 5B). The first region of interest occurs on the threshold between the weight-acceptance and standing phase of the lift, where the 50% and 60% MLC conditions had less flexion that the 80%, 90%, and 100% load conditions (Figures 5A, C). The larger main effect was seen in the lift-to-platform height and place-on-platform phases (second region of interest) (Figure 5B), where increased loads showed increased flexion can be seen across all load condition *post hoc* comparisons less than 70% MLC and between 80% vs. 100% MLC comparison (Figure 5C).

Analysis of the lower thoracic sagittal spine angles between load conditions indicate two significant regions of interest (11%–26%, *p* = 0.01; 48%–92%, *p* = 0.01) (Figure 6B). The first region of interest occurs on the threshold between the weight-acceptance and standing phase of the lift, the 50% and 60% MLC conditions have greater flexion than the 80%, 90%, and 100% conditions (Figures 6A, C). The larger main effect was seen in the lift-to-platform height and place-on-platform phases (second region of interest) (Figure 6B), where significant positive differences can be seen across all lighter loads (<70% MLC) and between 80% vs. 100% MLC conditions *post hoc* comparisons, with the exception of the 60% vs. 70% MLC comparison (Figure 6C).

Two significant regions of interest were shown in the analysis of the upper lumbar spine between load conditions (9%–30%, *p* = 0.01; 47%–88%, *p* = 0.01) (Figure 7B). During the weight-acceptance to standing phase of the lift (first region of interest) the 50% MLC condition has less flexion when compared all other conditions, while the 90% and 100% MLC conditions have greater flexion than the 60% and 70% conditions (Figure 7A). The larger main effect was seen in the lift-to-platform height and place-on-platform phases (second region of interest) (Figure 7B), where significant positive differences can be seen across all load conditions <80% MLC, except for 60% vs. 70% (Figure 7C).

The lower lumbar segment revealed only one region of interest in analysis (51%–79%, *p* = 0.01) spanning the lift-to-platform height and place-on-platform phases (Figure 8B). There was an increase in the 100% MLC extension when compared to the 50%–80% MLC conditions in *post hoc* comparison as well as the 50% vs. the 60%, 80%, and 90% MLC conditions (Figures 8A, C).

### 3.2 Discrete variable analysis

**FIGURE 9:**
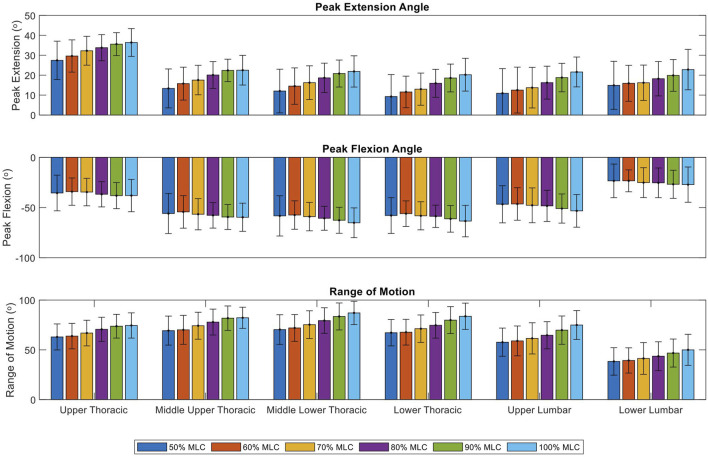
Peak extension, peak flexion and range of motion of six load conditions grouped by spinal segments. Bars represent the mean peak extension, flexion and range of motion angles. Lines at the bar ends indicate SD.

Using the knowledge gained from SPM analysis; two regions of interest are present in all segments of the spine. These occurred at an area of peak flexion and peak extension. Therefore, analysis focusing on peak extension, peak flexion and range of motion was performed to see if the relationships between load conditions were present in discrete variables (Figure 9).

Significant effect of load conditions on spine peak extension were found for the upper thoracic (F = 24.09, *p* < 0.001), middle upper thoracic (F = 24.73, *p* < 0.001), middle lower thoracic (F = 36.65, *p* < 0.001), lower thoracic (F = 52.04, *p* < 0.001), upper lumbar (F = 66.38, *p* < 0.001) and lower lumbar (F = 26.81, *p* < 0.001) (Table 1). Post-hoc tests revealed that with an increase in load condition, the peak extension of each segment significantly increases (*p* < 0.001), this trend is illustrated in Figure 9. The positive relationship was mainly present when comparing the lighter load conditions (50%–70% MLC) to the heavier load conditions (80%–100% MLC). While the upper thoracic segment has the largest peak extension for all load conditions, the change in peak extension due to an increase in loading does not vary much between segments with ranges between 7.8° to 10.9° (Figure 9; Table 1).

**TABLE 1:** Peak extension mean angles (°), standard deviations, and results of the repeated measures ANOVA for spine segments at the six load conditions.

	100 (a)	90 (b)	80 (c)	70 (d)	60 (e)	50 (f)	F value	*p*-value
Upper thoracic	36.4 ± 12.07^d,e,f^	35.6 ± 12.37^d,e,f^	33.8 ± 11.03^e,f^	32.3 ± 10.96^a,b,e,f^	29.6 ± 9.77^a,b,c,d,f^	27.4 ± 9.63^a,b,c,d,e^	24.09	**p < 0.001**
Middle upper thoracic	22.5 ± 9.03^d,e,f^	22.4 ± 11.52^d,e,f^	20.1 ± 7.93^e,f^	17.5 ± 9.16^a,b,f^	15.8 ± 8.26^a,b,c,f^	13.4 ± 8.09^a,b,c,d,e^	24.73	**p < 0.001**
Middle lower thoracic	21.9 ± 8.87^c,d,e,f^	20.8 ± 10.19^d,e,f^	18.7 ± 8.07^a,d,e,f^	16.2 ± 8.46^a,b,c,e,f^	14.5 ± 7.41^a,b,c,d,f^	12.0 ± 7.25^a,b,c,d,e^	36.65	**p < 0.001**
Lower thoracic	20.2 ± 8.63^c,d,e,f^	18.6 ± 8.28^d,e,f^	15.9 ± 6.99^a,d,e,f^	13.0 ± 7.36^a,b,c,f^	11.6 ± 6.75^a,b,c,f^	9.3 ± 6.57^a,b,c,d,e^	52.04	**p < 0.001**
Upper lumbar	21.6 ± 7.96^c,d,e,f^	18.8 ± 7.15^c,d,e,f^	16.2 ± 6.99^a,b,d,e,f^	13.7 ± 6.77^a,b,c,e,f^	12.5 ± 5.63^a,b,c,d,f^	10.9 ± 5.76^a,b,c,d,e^	66.38	**p < 0.001**
Lower lumbar	22.8 ± 10.15^c,d,e,f^	19.9 ± 7.50^d,e,f^	18.2 ± 8.24^a,d,e,f^	16.2 ± 7.84^a,b,c^	15.9 ± 7.45^a,b,c^	14.9 ± 6.98^a,b,c^	26.81	**p < 0.001**

Significant *p*-values (*α* = 0.05) are marked in bold. Post-hoc significant differences (*α* = 0.0033) are marked with a (vs. 100% MLC), b (vs. 90% MLC), c (vs. 80% MLC), d (vs. 70% MLC), e (vs. 60% MLC), f (vs. 50% MLC).

Significant effect of load conditions on spine peak flexion were found for the middle upper thoracic (F = 2.62, *p* = 0.027), middle lower thoracic (F = 5.15, *p* < 0.001), lower thoracic (F = 4.15, *p* = 0.0015), upper lumbar (F = 8.18, *p* < 0.001) and lower lumbar (F = 3.78, *p* = 0.0030) (Table 2). Post-hoc tests showed few results between the condition comparisons for the middle lower thoracic and upper lumbar spine segments. This relationship was only present when comparing the lightest load condition (50% MLC) to the heavier load conditions (60%–100% MLC). The middle lower thoracic segment has the greatest flexion of −65.2° but significant differences in load conditions peak flexion were not present for most comparisons. As illustrated in Figure 9 there is no clear positive or negative trend.

**TABLE 2:** Peak flexion mean angles (°), standard deviations, and results of the repeated measures ANOVA for spine segments at the six load conditions.

	100 (a)	90 (b)	80 (c)	70 (d)	60 (e)	50 (f)	F value	*p*-value
Upper thoracic	−38.1 ± 16.71	−38.2 ± 18.50	−36.8 ± 17.85	−34.6 ± 20.05	−34.2 ± 19.99	−35.5 ± 17.78	2.15	0.063
Middle upper thoracic	−59.7 ± 10.88	−59.4 ± 16.23	−57.8 ± 12.76	−56.7 ± 14.17	−54.3 ± 16.22	−56.0 ± 13.58	2.62	**0.027**
Middle lower thoracic	−65.2 ± 14.88^a^	−62.7 ± 17.36	−60.7 ± 14.00	−59.1 ± 14.11	−57.5 ± 15.53	−58.3 ± 13.68^f^	5.15	**p < 0.001**
Lower thoracic	−63.5 ± 14.78	−61.3 ± 15.52	−58.7 ± 11.09	−58.2 ± 11.91	−56.1 ± 12.70	−57.9 ± 12.60	4.15	**0.0015**
Upper lumbar	−53.3 ± 13.99^a^	−51.0 ± 14.51	−48.4 ± 13.27^a^	−47.8 ± 12.93^a^	−46.5 ± 12.43^a^	−46.8 ± 13.00^c,d,e,f^	8.18	**p < 0.001**
Lower lumbar	−27.2 ± 17.56	−26.9 ± 16.25	−25.4 ± 15.67	−25.2 ± 14.84	−23.4 ± 14.01	−23.5 ± 16.08	3.78	**0.0030**

Significant *p*-values (*α* = 0.05) are marked in bold. Post-hoc significant differences (*α* = 0.0033) are marked with a (vs. 100% MLC), b (vs. 90% MLC), c (vs. 80% MLC), d (vs. 70% MLC), e (vs. 60% MLC), f (vs. 50% MLC).

Significant effect of load conditions on spine ROM were found for the upper thoracic (F = 19.01, *p* < 0.001), middle upper thoracic (F = 27.91, *p* < 0.001), middle lower thoracic (F = 32.24, *p* < 0.001), lower thoracic (F = 29.96, *p* < 0.001), upper lumbar (F = 45.62, *p* < 0.001) and lower lumbar (F = 24.96, *p* < 0.001) (Table 3). Post-hoc tests exposed that with an increase in load condition, the ROM of each segment significantly increases (*p* < 0.001). This relationship is across all conditions with the exception of the 50% vs. 60% comparison for all spine segments and the 90% vs. 100% comparison for the upper thoracic and middle upper thoracic segments. The middle lower thoracic segment has the greatest range of motion for all load conditions, with its largest range of motion being 87° for the 100% MLC condition. A positive trend in range of motion can be seen in Figure 9.

**TABLE 3:** Range of motion mean angles (°), standard deviations, and results of the repeated measures ANOVA for spine segments at the six load conditions.

	100 (a)	90 (b)	80 (c)	70 (d)	60 (e)	50 (f)	F value	*p*-value
Upper thoracic	74.7 ± 13.63^d,e,f^	73.8 ± 13.93^d,e,f^	70.7 ± 13.02^d,e,f^	66.8 ± 14.64^a,b,c,e^	63.7 ± 14.35^a,b,c,d^	63.0 ± 13.05^a,b,c^	19.01	**p < 0.001**
Middle upper thoracic	82.3 ± 14.48^d,e,f^	81.7 ± 13.97^d,e,f^	77.8 ± 12.70^d,e,f^	74.0 ± 13.34^a,b,c,e,f^	69.8 ± 14.35^a,b,c,d^	69.3 ± 12.76^a,b,c,d^	27.91	**p < 0.001**
Middle lower thoracic	87.0 ± 15.69^c,d,e,f^	83.5 ± 15.40^d,e,f^	79.3 ± 13.58^a,d,e,f^	75.1 ± 13.73^a,b,c,e,f^	71.7 ± 13.42^a,b,c,d^	70.4 ± 12.91^a,b,c,d^	32.24	**p < 0.001**
Lower thoracic	83.7 ± 14.23^c,d,e,f^	79.9 ± 13.37^c,d,e,f^	74.7 ± 12.68^a,b,d,e,f^	71.1 ± 12.62^a,b,c,e^	67.6 ± 12.80^a,b,c,d^	67.22 ± 12.12^a,b,c^	29.96	**p < 0.001**
Upper lumbar	75.0 ± 13.86^b,c,d,e,f^	69.9 ± 13.97^a,c,d,e,f^	64.7 ± 13.35^a,b,d,e,f^	61.6 ± 13.31^a,b,c^	59.0 ± 12.02^a,b,c^	57.7 ± 11.92^a,b,c^	45.62	**p < 0.001**
Lower lumbar	49.3 ± 15.78^c,d,e,f^	46.3 ± 14.49^d,e,f^	43.1 ± 13.27^a,e,f^	40.9 ± 11.78^a,b^	38.9 ± 10.74^a,b,c^	38.4 ± 12.68^a,b,c^	24.96	**p < 0.001**

Significant *p*-values (*α* = 0.05) are marked in bold. Post-hoc significant differences (*α* = 0.0033) are marked with a (vs. 100% MLC), b (vs. 90% MLC), c (vs. 80% MLC), d (vs. 70% MLC), e (vs. 60% MLC), f (vs. 50% MLC).

## 4 Discussion

This study aimed to describe the effects of increased loading conditions on spinal angles at six points along the spine using small, portable, wireless sensors using SPM. It was hypothesised that there with an increase in load there would be a decrease in spinal flexion occurring during the weight-acceptance phase and an increase in spinal extension occurring during the lift-to-platform-height phase. SPM results showed that there are two regions of interest that occur during the lift, around the point of peak flexion which occurred during the transition from the weight-acceptance to standing phases and during peak extension which occurred in the transition between the lift-to-platform-height and the place-on-platform phases. Therefore, the regions of interest occurred later in the lift than was hypothesised. Post-hoc analysis revealed that there few differences between load conditions occurring during the period of peak flexion, however during the period of peak extension statistically significant differences between load conditions were seen for the majority of the lift from the lift-to-platform-height phase to the end of the lift.

This research found a minimal relationship between increased load and an increase in peak flexion, occurring only in the middle upper thoracic and upper lumbar segments. Previous studies have shown a trend of increased peak flexion with and increase in load for the lumbar spine (Davis and Marras, 2000; Melino et al., 2014; Chowdhury et al., 2018) while others found no significant differences (Allread et al., 1996; Song and Qu, 2014b; Antwi-Afari et al., 2018; West et al., 2018). The significance found in these studies could be because they used heavier loads with Davis and Marras (2000) maximal load of 41.8 kg and Melino et al. (2014) using a safe maximal lift (>20 kg). Or in the case of Chowdhury et al. (2018) it could due to studying multiple lumbar segments (L2/L3, L3/L4, L5/S1). While the studies reporting no-significant results used relatively light load conditions (<16 kg), with the exception of MacKinnon and Li (1998) with a maximal load of 66% of bodyweight but a sample size of five males. The small range in peak flexion from lighter to heavier load conditions may be due to participants trying to maintain an upright position of the torso in order to get lower in the squat position to transfer the heavy loads closer to the body’s midline.

The peak extension angle for the 100% and 90% MLC conditions were consistantly different to the lighter 70%–50% MLC conditions, with a reduction in peak extension angle with a decrease in load. Peak extension angles consistantly increased when the load condidtion increased in all spine segments. Significant differences in lumbar peak extension were found in previous research (Allen et al., 2012; Melino et al., 2014; Chowdhury et al., 2018), other research reported no differences due to increased loading (Faber et al., 2009; Song and Qu, 2014b; Antwi-Afari et al., 2018; West et al., 2018). However, the studies reporting no differences were comparing loading conditions at a single lumbar spine point (L5/S1) or the spine as a single trunk segment. This loss of detail in the study of the spine could contribute to the lack of results. The peak extension was largest in the upper thoracic segment, this could be due to the need to counter-balance the load when it reaches the peak in its trajectory at the completion of the lift-to-platform-height phase.

The 100%–80% MLC conditions were consistently different to that of the 70%–50% conditions showing an increase in spine segment ROM with an increase in load condition. This research is quantitatively comparable to previous research that found significant increases in lumbar ROM with an increase in load conditions. Lumbar ROM was reported as 39.43° at a 10.2 kg load (Allread et al., 1996) and at 35.4° at 5 kg load (Conforti et al., 2020) to this researches lightest condition at 38.4°. Some previous research also found spine ROM to have no significant differences due to load (Granata et al., 1999; Antwi-Afari et al., 2018).

Difference in the spine angles due to the load conditions do not occur at a single time point during the lift, spanning over regions of the lift. Looking at only select spine segments and/or discrete variables/time points does not allow the exploration along the entire spine and its trajectory for the duration of the lift and would not provide the detail to see where, how and when each spine segment is affected by the increased loads. For example, many studies reported significant differences for loading conditions at specific time points, such as the start and end frames of the lift (Allen et al., 2012; Song and Qu, 2014a; Song and Qu, 2014b; West et al., 2018). However, in this research at the start and end frames of the lift there were no significant results. It is therefore beneficial to look at the complete duration of a task to determine regions of interest for analysis, Injurious lifting motions have been attributed to hyperextension of the spine (Neumann, 2009). Analysis of the spine angles for the whole lift showed that all spine segments entered a phase of extension beyond neutral. However, Allen et al. (2012), West et al. (2018) found a decrease in angle (less extension) when analysing the thoracic spine at C7–T7 (upper thoracic to middle upper thoracic) and an increase in angle for the lumbar spine (L5) (Allen et al., 2012), this is in contrast with our findings. In this study the upper thoracic segment had the largest extension angle (36.4°), while all inferior segments peak extension angle was between 20.2–22.8°. These studies were a good comparison as they also performed minimum and maximum lifts to shoulder (or above) height, a similar procedure used in these experimental trials. With the upper thoracic segment entering a phase of greater extension than other segments during the lift-to-height and place-on-platform stages of the lift, it could be at an increased risk of injury. Knowing how and when increased load impacts individual spine segments is crucial for understanding the spine’s compensatory mechanisms. This knowledge could inform the development of assistive devices by targeting when and where assistance is most needed.

In future work, this research intends to use the knowledge gathered and spine angle data collected to create generalised machine learning models able to predict whether a lift is heavy or light based on a maximum lift capacity threshold. In simple terms, whether a load is within a safe range to lift. The predictive model could then be applied to a wearable smart assistive device that would be capable of providing real-time user feedback or augmentation. The 100%–80% MLC conditions were shown to be consistently significantly different from the other loading conditions and in previous research looking at the relationship between MLC and maximum acceptable lift (maximum load that can be comfortably lifted), it was found that the maximum acceptable weight of a lift was 84% ± 8% of MLC (Savage et al., 2012). New technologies such as machine learning thrive in making predictions without needing to be explicitly told the relationship between the variables (Bzdok et al., 2018) and have been shown to perform well for task classification using biomechanical datasets (Bagnall et al., 2017).

## 5 Conclusion

This study has shown that the effect of an increase in load on spine kinematics, at all levels of the spine, is evident when comparing the multi-segmental spine angles during a ground-to-platform manual handling task. Two regions of interest were present in the time series analysis. During the transition from the weight-acceptance to standing phase, which involved a period of peak flexion and during the transition from the lift-to-platform-height to the place-on-platform phase, which involved a period of peak extension. As the 100%–80% MLC load conditions significantly increase spinal peak extension and ROM this is a good indication that predictive models will be able to accurately classify light vs. heavy loads. Additionally, knowing that there are two regions of a lifting task where an increase in load affects spinal angles means that smart assistive devices should be targeting these specific transition periods for injury preventative support. Both of these factors are key knowledge in informing the design of smart targeted assistive devices.

### 5.1 Limitations

The lift height (platform of 1.4 m) was not normalised to participant height and therefore the height of the participant may have an effect on the resulting spine angles, however a set height was used as ground to platform lifting tasks in the workplace would not be adjusted to an individual’s height. Additionally, the experiments were conducted in a controlled laboratory setting, environmental factors (e.g., temperature, ground surface) may have an effect on manual handling personnel that future work could take into account. This research only represents angles in the sagittal plane as this is the direction where the majority of lifting angles occurs, however motion would still be present in the transverse and coronal plane that would contribute to lifting and these contributions could be studied in future work. Participants completed up to ten submaximal lifts prior to reaching their 100% MLC load, it is therefore possible that this would be slightly under their true maximum, however a gradual increase in load was believed to be the safest approach to determining MLC.

## Data Availability

The raw data supporting the conclusion of this article will be made available by the authors, without undue reservation.
